# Geographic Variation in Hospital-Based Physician Participation in Insurance Networks

**DOI:** 10.1001/jamanetworkopen.2022.15414

**Published:** 2022-05-31

**Authors:** Sayeh Nikpay, Leonce Nshuti, Michael Richards, Melinda B. Buntin, Daniel Polsky, John A. Graves

**Affiliations:** 1 Department of Health Policy and Management, University of Minnesota School of Public Health, Minneapolis; 2 Department of Health Policy, Vanderbilt University School of Medicine and Vanderbilt University Medical Center, Nashville, Tennessee; 3 Department of Economics, Baylor University, Waco, Texas; 4 Department of Health Policy and Management, Bloomberg School of Public Health and Carey Business School, Johns Hopkins University, Baltimore, Maryland

## Abstract

This cross-sectional study documents 2021 insurance network participation rates among hospital-based physicians nationally and by state.

## Introduction

“Surprise” bills occur when a patient receives an unexpected out-of-network bill, often after treatment at an in-network facility. Although many states limit this practice, the 2021 No Surprises Act provides consumers with federal protection that restricts surprise billing for insurance plans in states without restrictions and for large group insurance plans previously exempted from state-level protections. As of January 2022, unless stronger state-level patient protections are already in place, out-of-network clinicians must accept insurers’ median in-network payment rate or subject reimbursement decisions to an independent dispute resolution process.

Prior research has found that surprise billing practices were concentrated among certain hospital-based specialties (eg, emergency medicine and anesthesiology).^1,2,3^ However, the degree to which insurance network participation varies within and across specialties and states remains unknown. Investigating this variation is important to understand where reductions in surprise billing could occur. Moreover, while surprise billing protections are intended to reduce financial incentives to balance bill, the option of out-of-network reimbursement at median in-network rates may dilute insurer and clinician incentives to negotiate insurance network contracts, resulting in narrower networks. Therefore, to establish a baseline against which changes in insurance network participation can be assessed, this study documented 2021 insurance network participation rates among hospital-based physicians nationally and by state.

## Methods

This descriptive, cross-sectional study used 2021 data from Vericred on 881 insurance networks associated with fully insured small-group and individually purchased plans available in the Patient Protection and Affordable Care Act’s marketplaces.^4^ We pooled data from Physician Compare, IQVIA, and the National Plan and Provider Enumeration System to cross-validate information on specialty and geographic location for anesthesiologists, emergency medicine physicians, and surgeons (general surgeons and those with a primary specialty in colon or rectal surgery, head and neck surgery, neurosurgery, orthopedics, thoracic surgery, transplants, or trauma surgery).^5^

To identify hospital-based physicians, we restricted our sample to those with practice locations within 500 m of an acute-care hospital. Our primary measure was the percentage of physicians who accepted any insurance plan network. We calculated this percentage by specialty and, within specialties, by state. Our results cover only the continental United States, as some of our input data sources lacked comprehensive physician-specific data for Alaska and Hawaii. Our results also classified states by whether they had existing comprehensive balance billing protections prior to 2022.^6^ Our study followed the Strengthening the Reporting of Observational Studies in Epidemiology (STROBE) reporting guideline and was deemed exempt by the institutional review board at Vanderbilt University Medical Center because no patient data were used and all physician-level data were publicly accessible via Physician Compare and the National Plan and Provider Enumeration System.

## Results

Our final sample included 25 739 anesthesiologists, 22 972 emergency medicine physicians, and 23 395 surgeons from 48 states (Table). Nationally, 85.4% of anesthesiologists, 82.6% of emergency medicine physicians, and 97.4% of surgeons participated in at least 1 private health plan network. Among anesthesiologists and emergency medicine physicians, there was wide variation by state. In Iowa, 98.4% of emergency medicine physicians accepted at least 1 network, while in North Carolina, 51.8% accepted at least 1 network. Among surgeons, there was less variation, with the lowest participation rate observed in New Jersey (93.7%).

**Table.** Data on Hospital-Based Physicians Participating in at Least 1 Insurance Plan Network^a^

State	Physicians, No./total No. (%)		
	Anesthesiology	Emergency medicine	Hospital-based surgery
All	21 975/25 739 (85.4)	18 969/22 972 (82.6)	22 777/23 395 (97.4)
Alabama	187/325 (57.5)	174/232 (75.0)	319/322 (99.1)
Arizona	274/304 (90.1)	386/418 (92.3)	397/403 (98.5)
Arkansas	116/117 (99.1)	160/166 (96.4)	146/146 (100.0)
California^b^^,^^c^	3054/3251 (93.9)	2502/2968 (84.3)	2615/2764 (94.6)
Colorado^b^^,^^c^	288/418 (68.9)	146/226 (64.6)	404/411 (98.3)
Connecticut^b^	310/324 (95.7)	302/348 (86.8)	230/232 (99.1)
Delaware	28/36 (77.8)	33/44 (75.0)	37/37 (100.0)
Florida^b^^,^^c^	686/1244 (55.1)	683/1253 (54.5)	1002/1024 (97.9)
Georgia^b^^,^^c^	611/657 (93.0)	601/680 (88.4)	768/785 (97.8)
Idaho	64/64 (100.0)	78/78 (100.0)	103/103 (100.0)
Illinois^c^	772/1065 (72.5)	771/995 (77.5)	879/895 (98.2)
Indiana	640/708 (90.4)	413/491 (84.1)	477/483 (98.8)
Iowa	257/259 (99.2)	240/244 (98.4)	313/318 (98.4)
Kansas	174/175 (99.4)	167/170 (98.2)	207/208 (99.5)
Kentucky	245/308 (79.5)	232/266 (87.2)	352/361 (97.5)
Louisiana	233/260 (89.6)	335/418 (80.1)	372/382 (97.4)
Maine^b^^,^^c^	124/143 (86.7)	182/195 (93.3)	158/160 (98.8)
Maryland^b^	523/573 (91.3)	210/257 (81.7)	433/438 (98.9)
Massachusetts	935/966 (96.8)	686/763 (89.9)	751/793 (94.7)
Michigan^b^^,^^c^	566/733 (77.2)	722/977 (73.9)	628/635 (98.9)
Minnesota	388/424 (91.5)	352/369 (95.4)	474/474 (100.0)
Mississippi	134/142 (94.4)	160/192 (83.3)	194/196 (99.0)
Missouri	535/551 (97.1)	540/577 (93.6)	515/524 (98.3)
Montana	173/174 (99.4)	91/91 (100.0)	154/156 (98.7)
Nebraska	104/106 (98.1)	54/54 (100.0)	159/160 (99.4)
Nevada	80/95 (84.2)	128/185 (69.2)	139/142 (97.9)
New Hampshire^c^	159/161 (98.8)	116/118 (98.3)	162/163 (99.4)
New Jersey^c^	775/800 (96.9)	497/585 (85.0)	385/411 (93.7)
New Mexico^b^	116/137 (84.7)	187/195 (95.9)	165/171 (96.5)
New York^c^	2061/2180 (94.5)	1275/1345 (94.8)	1265/1311 (96.5)
North Carolina	349/720 (48.5)	368/710 (51.8)	748/765 (97.8)
North Dakota	66/66 (100.0)	27/27 (100.0)	75/75 (100.0)
Ohio^b^^,^^c^	688/955 (72.0)	730/998 (73.1)	882/891 (99.0)
Oklahoma	176/299 (58.9)	152/243 (62.6)	274/281 (97.5)
Oregon^b^	215/260 (82.7)	289/331 (87.3)	411/415 (99.0)
Pennsylvania	1144/1179 (97.0)	931/990 (94.0)	1041/1064 (97.8)
Rhode Island	90/90 (100.0)	143/156 (91.7)	120/122 (98.4)
South Carolina	337/340 (99.1)	308/343 (89.8)	352/355 (99.2)
South Dakota	66/66 (100.0)	44/44 (100.0)	90/90 (100.0)
Tennessee	260/397 (65.5)	265/376 (70.5)	487/496 (98.2)
Texas^c^	1653/1666 (99.2)	1255/1304 (96.2)	1525/1567 (97.3)
Utah	152/247 (61.5)	125/230 (54.3)	195/199 (98.0)
Vermont	86/90 (95.6)	46/46 (100.0)	91/91 (100.0)
Virginia^c^	493/643 (76.7)	428/582 (73.5)	515/519 (99.2)
Washington^c^	734/899 (81.6)	656/688 (95.3)	696/724 (96.1)
West Virginia	54/90 (60.0)	99/143 (69.2)	148/156 (94.9)
Wisconsin	446/605 (73.7)	390/488 (79.9)	481/484 (99.4)
Wyoming	54/58 (93.1)	66/69 (95.7)	64/64 (100.0)

^a^ Analysis of 2021 Vericred network data for fully insured individual and small-group plans available in the Patient Protection and Affordable Care Act marketplaces.

^b^ State has payment standard as part of existing comprehensive surprise billing protections.

^c^ State has dispute resolution process as part of existing comprehensive surprise billing protections.

## Discussion

This study documented variation in insurance network participation among hospital-based physicians and, within those specialties, across states. In some states, a large percentage of emergency medicine physicians and anesthesiologists did not participate in any insurance network, whereas hospital-based surgeon participation was high (>90%) in all states. Limitations to this study include possible miscategorization of hospital-based and clinic-based physicians and inaccuracies in physician network data. However, we found similar results in sensitivity analyses that were not restricted to physicians located within 500 m of a hospital, and inaccuracies would affect our results only if a given physician was missing from every network in our sample.

Our results suggest that reductions in surprise billing will vary across states. Moreover, because clinicians and insurers may face diluted incentives to negotiate in-network payment rates when a default payment level for out-of-network care applies (eg, median in-network rate), participation in insurance networks could decline. For nonemergency services, the net effect may be a reduction in surprise bills but an increase in out-of-network patient cost sharing owing to narrower networks. Our results provide a baseline from which these changes can be assessed.
